# Awareness Regarding Venous Thromboembolism and Pulmonary Embolism After Pregnancy and Cesarean Section in Female Population in the Aseer Region, Saudi Arabia

**DOI:** 10.7759/cureus.51272

**Published:** 2023-12-29

**Authors:** Javed Iqbal Wani, Mir Nadeem

**Affiliations:** 1 Internal Medicine, King Khalid University Hospital, Abha, SAU; 2 General Internal Medicine, The Royal Wolverhampton NHS Trust, Wolverhampton, GBR

**Keywords:** healthcare worker awareness, pulmonary infarction, pulmonary vascular disease, deep vein thrombosis (dvt), lung embolus

## Abstract

Introduction

A pulmonary embolism (PE) occurs when an embolus that has traveled through the venous system from another part of the body obstructs an artery in the lungs. Chest pain, especially while breathing in, coughing up blood, and shortness of breath are all possible signs of PE. There could also be signs of a blood clot in the leg, like a painful, swollen, red, and warm leg. As a high-risk group, particularly during childbearing age, the aim of this study is to evaluate the general awareness of females regarding PE and identify areas of knowledge deficit and factors contributing to their awareness level.

Methods

A cross-sectional descriptive survey of Saudi women in general over the age of 18 was carried out. Participants were asked to respond to a structured questionnaire that was used to gather data. The questionnaire was formulated in Google Forms with an Arabic translation of the form and the link generated and was sent to each participant for completion. In total, 827 respondents filled out the survey with accurate and complete information.

Results

The study comprised 827 female volunteers, with a mean age of 33.2 ± 9.4 years, ranging in age from 15 to 60. Additionally, 52.8% of the female sample had graduated from college, compared to about 4% who were illiterate. In general, 40.2% of the girls knew everything there was to know about PE.

Conclusions

According to the study's findings, the public female population knew less about PE overall - that is, about risk factors, symptoms, and preventive measures. As more knowledge about the dangers, causes, prevention, diagnosis, and treatment of PE becomes available, it is imperative that healthcare professionals translate and actively distribute this information to the public, particularly to women.

## Introduction

Pulmonary embolism (PE) is the occlusion of a lung artery by an embolus that has traveled through the venous system from another part of the body [1]. Chest pain, especially while breathing in, coughing up blood, and shortness of breath are all possible signs of PE [2]. A red, warm, swollen, and painful leg is a possible sign of a blood clot in the leg [3]. Rapid breathing, a fast heartbeat, low blood oxygen levels, and perhaps low-grade fever are symptoms of PE [4]. Severe cases may result in abrupt death, abnormally low blood pressure, and passing out [5]. PE affects an estimated 650,000 people [6]. About 200,000 deaths annually are attributed to this illness, which accounts for 15% of all hospital deaths [6]. It is estimated that 110,000 hospital admissions annually among persons over 65 in the United States are attributable to PE and its primary cause, deep vein thrombosis (DVT) [7]. Knowledge of the risk factors, prevention, and acute and long-term management of venous thromboembolism (VTE) has grown significantly within the past 20 years. This condition, which includes PE and DVT, is the third most common vascular diagnosis in the United States, behind myocardial infarction and stroke. It accounts for at least 3.2% of all cardiovascular-related deaths [8]. The majority of PE events are thought to be provoked; they are linked to triggering events such as hospitalization, surgery, trauma, immobilization, and cancer. Provoked events mostly affect women using oral contraceptives, postmenopausal estrogen, or pregnancy. Since there is a significant danger of embolism from the triggers, prophylactic medication is frequently employed. Chronic kidney illness, mild injuries, and inflammatory bowel disease are less well-known risk factors. The combination of the risk factors multiplicatively or additively increases the risk of VTE. For instance, the risk of VTE is doubled in obese women who use oral contraceptives, whereas the risk is tenfold higher in obese women who do not use oral contraceptives [9-11].

The current study was conducted to assess the general female population's awareness regarding PE, as they are a high-risk group during childbearing age with pregnancy, surgery, and obesity, increasing the risk of VTE or PE in them. Hence, the purpose is to detect areas of awareness deficit and factors contributing to their awareness level.

## Materials and methods

A descriptive cross-sectional study was conducted in the Aseer region of Saudi Arabia from December 2021 to January 2023 with a duration of 13 months.

Inclusion criteria: The study included all females above 18 years of age, with more preference for women of childbearing age defined as per the WHO definition of childbearing age.

Exclusion criteria: Females less than 18 years of age were excluded, as well as females who lacked the mental capacity to answer the questionnaire.

Data were collected using a well-structured online questionnaire to be answered by participants. The questionnaire was formulated in Google Forms with an Arabic translation of the form and the link generated and was sent to each participant for completion. The questionnaire was developed and reviewed by the researchers after an intensive literature review and after consulting experts in the research area, and any modifications were considered. The first part of the questionnaire included an introduction and instructions. The second part was about socio-demographics, including age, gender, level of education, nationality and occupation, and obstetric and family history. The third part was carefully designed to ask about the knowledge of PE covering causes, risk factors, signs, and preventive measures. A total of 827 participants completed the questionnaire online without missing or incorrect data.

The Institutional Review Board of King Khalid University approved this study under the ethical clearance number (HA-06-C-003), and this research was carried out in compliance with committee regulations.

Statistical analysis: Following collection, data were edited, coded, and entered into Statistical Product and Service Solutions (SPSS version 20; IBM SPSS Statistics for Windows, Armonk, NY), a statistical program. The provided graphs were created with Microsoft Excel. All statistical analyses were conducted with an alpha error of 0.05 and two-tailed testing. A P value of less than 0.05 was deemed statistically noteworthy. The exact Fishers test and the chi-square/Mont Carlo exact test were performed to examine any relationships between various female variables and awareness levels. Precise tests were employed if there are tiny frequencies where the chi-square is invalid. A multivariate logistic regression model was used to evaluate the adjusted relationship between the characteristics of females and their knowledge of PE.

## Results

The study included 827 female participants whose ages ranged from 15 years to 60 years old, with a mean age of 33.2 ± 9.4 years. About 4% of the sampled females were illiterate, while 52.8% of them were university graduates. Urban residence was recorded for 76.3% of the females. As for parity, 11.5% of the respondents were nulliparous, while 47.9% had two to four deliveries. Family history of DVT was recorded among 29.3% of the females, while family history of PE was recorded among 25.2% of them. The combined family history of DVT/PE was 15.8 among the participants. About 57% of the females were free of any chronic health problem, while 16.1% were diabetic and 6.5% had blood-clotting disorder. Considering the last pregnancy complications, 59.7% had no complications recorded, while 14% recorded dehydration, and 4.5% recorded varicose veins (Table 1).

**Table 1:** Bio-demographic characteristics of the general female population in the Aseer region, Saudi Arabia

Bio-demographic data		No	%
Age in years	25-29	298	36.0%
	30-39	324	39.2%
	40-49	151	18.3%
	50+	54	6.5%
Education	Non educated	31	3.7%
	Basic education	92	11.1%
	Secondary education	267	32.3%
	University	437	52.8%
Residence	Rural	196	23.7%
	Urban	631	76.3%
Parity	Nulliparous	95	11.5%
	Primipara	148	17.9%
	2-4	396	47.9%
	5+	188	22.7%
Family history of DVT	No	585	70.7%
	Yes	242	29.3%
Family history of pulmonary embolism	No	619	74.8%
	Yes	208	25.2%
DVT history	Yes	156	18.9%
	Maybe	671	81.1%
Chronic diseases	No	469	56.7%
	DM	133	16.1%
	Blood clotting	54	6.5%
	Anemia	60	7.3%
	GIT disorders	61	7.4%
	Varicose vein	50	6.0%
Last pregnancy complications	No	494	59.7%
	Severe dehydration	117	14.1%
	Anemia	82	9.9%
	Bleeding	63	7.6%
	Pre-eclampsia	34	4.1%
	Varicose vein	37	4.5%

As for awareness regarding PE (Table 2), 40.2% of the participant females had awareness about PE, and 30.5% could roughly define it correctly. Considering risk factors, prolonged immobility was recorded by 69.5% of the females, followed by surgery (65.9%), obesity (65.2%), smoking (62.2%), previous PE, and pregnancy (58.8% for each). Considering the causes of PE, 69.3% of the participants mentioned DVT or a clot in the leg, while 62.3% mentioned lower limb varicose veins. Considering PE signs, the inability to breathe was recorded by 78.1% of the participants, followed by acute chest pain (76.2%), hemoptysis (69.8%), and imbalance and drowsiness (67.7%). As for complications of PE, about 73% of the females mentioned pulmonary hypertension, and 71.9% recorded pulmonary injury. Concerning methods to prevent PE before surgery, 79% of the participants agreed on administrating anti-thrombotic drugs, and 70.9% recorded leg elevation after surgery. As for general procedures to prevent PE, 81.6% recorded frequent movement after long-standing, and 78.6% of the participants told about frequent fluid intake and leg messages (76.7%). Generally, 58% of the females had good awareness regarding all aspects of PE.

**Table 2:** PE awareness of the general female population in the Aseer region, Saudi Arabia

Domain	Knowledge items	No	%
General			
	Wrong answer/Don't know	575	69.5%
	Correct answer	252	30.5%
PE risk factors	Previous PE	486	58.8%
	Cancers	359	43.4%
	Cardiac disorders	461	55.7%
	Surgery	545	65.9%
	Prolonged immobility	575	69.5%
	Smoking	514	62.2%
	Obesity	539	65.2%
	Pregnancy	486	58.8%
Causes of PE	LL varicose vein	515	62.3%
	Dehydration	453	54.8%
	DVT	573	69.3%
Signs of PE	Inability to breath	646	78.1%
	Acute chest pain	630	76.2%
	Hemoptysis	577	69.8%
	Fever	538	65.1%
	Diziness	560	67.7%
Complications of PE	Pulmonary injury	595	71.9%
	Pulmonary hypertension	603	72.9%
	Heart failure	555	67.1%
Preventive methods of PE with surgery	thromboprophylaxis before and after surgery	654	79.1%
	Tight socks on the limb	500	60.5%
	Leg elevation after surgery	586	70.9%
General preventive methods of PE	Frequent fluid intake	650	78.6%
	Frequent movement in between long-standings	675	81.6%
	Wear suitable socks	618	74.7%
	Leg massage	634	76.7%
Signs of varicose vein	Foot and leg pain	587	71.0%
	LL edema	596	72.1%
	Leg redness	632	76.4%
	Leg hotness	561	67.8%

On relating females' awareness to their characteristics (Table 3), it was clear that 55.6% of old-aged females had a good awareness level compared to 54% of young-aged participants (P=0.015). As for education, 60% of university-graduated females had a good awareness level compared to 25.8% of non-educated females, with recorded statistical significance (P=0.001). Considering parity, 68.6% of multiparous females (five or more) recorded a good awareness level compared to 46.3% of the nulliparous group (P=0.001). About 60% of those who had a DVT history were highly knowledgeable compared to none of those who had not (P=0.001). Additionally, 69.7% of those who heard about PE had a good awareness level compared to 50.2% of those who did not (P=0.001).

**Table 3:** Relation between the bio-demographic data of the population and their awareness level regarding PE in the Aseer region, Saudi Arabia *Statistical significance

Bio-demographic data		Overall PE awareness				P value
		Poor		Good		
		No	%	No	%	
Age in years	25-29	137	46.0%	161	54.0%	0.015*
	30-39	140	43.2%	184	56.8%	
	40-49	46	30.5%	105	69.5%	
	50+	24	44.4%	30	55.6%	
Education	Non-educated	23	74.2%	8	25.8%	0.001*
	Basic education	44	47.8%	48	52.2%	
	Secondary education	105	39.3%	162	60.7%	
	University	175	40.0%	262	60.0%	
Residence	Rural	80	40.8%	116	59.2%	0.711
	Urban	267	42.3%	364	57.7%	
Parity	Nulliparous	51	53.7%	44	46.3%	0.001*
	Primipara	71	48.0%	77	52.0%	
	2-4	166	41.9%	230	58.1%	
	5+	59	31.4%	129	68.6%	
Family history of DVT	No	233	39.8%	352	60.2%	0.054
	Yes	114	47.1%	128	52.9%	
Family history of pulmonary embolism	No	257	41.5%	362	58.5%	0.685
	Yes	90	43.3%	118	56.7%	
DVT history	No	0	0.0%	0	0.0%	0.001*
	Yes	88	56.4%	68	43.6%	
	Maybe	259	38.6%	412	61.4%	
Chronic diseases	No	167	35.6%	302	64.4%	0.069
	Yes	180	50.3%	178	49.7%	
Could roughly define PE and VTE	No	246	49.8%	248	59.8%	0.001*
	Yes	101	30.3%	232	40.2%	

Finally, regression analysis for adjusted relation revealed that, among all included predictors, awareness level, high education, parity, DVT history, chronic disease, and hearing about PE were the most important significant predictors after fixing all other factors (Table 4). As for education, highly educated females recorded a 34% higher awareness level than others (OR=1.34; 95% CI: 1.10-1.64). The increased parity was associated with an increased likelihood of improved awareness by 47% than the low parity group (OR=1.47; 95% CI: 1.2-1.8). Females with DVT history recorded a doubled level of awareness compared to those without (OR=2.12; 95% CI: 1.36-3.31). In addition, those who previously heard about PE had as twice as level of awareness compared to those who did not (OR=2.53, 95% CI: 1.84-3.49).

**Table 4:** Multiple logistic regression analysis of the predictors for awareness regarding PE among the female population in the Aseer region

Predictor	B	S.E.	Sig.	OR	95% C.I. for OR	
					Lower	Upper
Age in years	0.00	0.10	0.981	1.00	0.82	1.23
High education	0.30	0.10	0.004	1.34	1.10	1.64
Urban residence	-0.31	0.19	0.102	0.73	0.50	1.06
Parity	0.38	0.10	0.001	1.47	1.20	1.80
DVT FH	-0.11	0.18	0.549	0.90	0.63	1.28
PE FH	0.14	0.19	0.469	1.15	0.79	1.67
DVT History	0.75	0.23	0.001	2.12	1.36	3.31
Chronic disease	-0.52	0.17	0.002	0.59	0.43	0.83
Know about PE	0.93	0.16	0.001	2.53	1.84	3.49
Constant	-2.29	0.65	0.001	0.10		
Model pseudo R^2^; (P)	15%; (0.001)					
Model accuracy	67%					

## Discussion

VTE remains one of the leading causes of maternal death and the most ignored medical complication during pregnancy and after the cesarian section. The pathophysiological changes that occur during pregnancy create a prothrombotic state in the body, increasing the basal risk, and it requires risk stratification to determine those who will derive the greatest benefit from thromboprophylaxis [12] among the risk factors of VTE among pregnant women; cesarean section is the most prevalent risk factor among the study participants, followed by obesity and multiparty [13]. To tackle the problem, awareness about the disease is the initial step to be taken, and in our study, we have briefly tried to root out awareness among females. The results of our study showed low levels of awareness and knowledge about VTE and its manifestations (DVT and PE), risk factors, and symptoms among women in the Aseer region, as was seen by Alzoubi et al. [14]. However, our findings were contrary to those of Kingman et al. [15]. Studies on patients’ awareness of VTE in pregnancy and after a cesarean section are limited. Our study reports that 40.2% of females had awareness about VTE and PE, and 30.5% defined it correctly, as seen by Alzoubi et al. [14] who showed that 46% of females in their study population had awareness. However, our results were not in accordance with those of Sarah et al. [16] who found good awareness among females in Jeddah province. Considering risk factors, prolonged immobility was recorded by 69.5% of the females, followed by surgery (65.9%), obesity (65.2%), smoking (62.2%), previous PE, and pregnancy (58.8% for each), as was seen by the study by Le Sage et al. [17]. Regarding the signs and symptoms of VTE and PE, the inability to breathe was recorded by 78.1% of the participants, followed by acute chest pain (76.2%), hemoptysis (69.8%), and imbalance and drowsiness (67.7%). Although the overall knowledge about VTE was less, the signs and symptoms were correctly identified by the females in the questionnaire as the warning signs in pregnancy that can relate to embolism or a serious health issue, as seen by Le Sage et al. [17].

As for education, 60% of university-graduated females had a good awareness level compared to 25.8% of non-educated females with recorded statistical significance (P=0.001). These findings were related to more use of social media and internet services to get more knowledge. It was also found that females who were educated by medical professionals had more knowledge and clear ideas about the risk factors and signs and symptoms of VTE and PE in pregnancy and post-caesarean section. These findings were statistically significant and as per previous studies [14].

On relating females' awareness to their characteristics, it was clear that 55.6% of old-aged females had a good awareness level compared to 54% of young-aged participants (P=0.015). Considering parity, 68.6% of multiparous females (five or more) recorded a good awareness level compared to 46.3% of the nulliparous group (P=0.001), possibly related to their less exposure with patient education in comparison with that of older patients who have multiple visits and pregnancy-related admissions to hospital. These findings paralleled those of Alzoubi et al. [14].

About 60% of those who had a DVT history were highly knowledgeable compared to none of those who had not (P=0.001). Moreover, 69.7% of those who heard about PE had a good awareness level compared to 50.2% of those who did not (P=0.001). These findings give very important weightage to hospital education in primary care level, maternity, and prenatal clinics about VTE and PE in pregnancy and post-surgery.

Our study had limitations as it was a questionnaire-based and no intervention-based study, and patients' self-reporting method was used, which undesirably suffered recall and social desirability bias; however, we followed the most widely used method in survey studies.

## Conclusions

There is less awareness about VTE and pulmonary embolism in pregnancy among females of the Aseer region of Saudi Arabia. Hence, the necessity for patient education and the creation of community-level awareness activities should be highlighted.

Primary care physicians can significantly lower patient mortality and morbidity by educating their patients, making early diagnoses, and providing care. Healthcare professionals play a crucial role in educating pregnant women about VTE and its symptoms, indicators, and inpatient and outpatient maternity wards. More effort has to be made at the hospital and primary care physician levels to better inform females about these issues.
